# Late Pleistocene-Holocene sedimentary evolution in the coastal zone of the Red River Delta

**DOI:** 10.1016/j.heliyon.2020.e05872

**Published:** 2021-01-19

**Authors:** Hoang Phan Hải Yen, Tran Thị Thanh Nhan, Tran Nghi, Ngo Quang Toan, Hoang Anh Khien, Doan Dinh Lam, Hoang Van Long, Dinh Xuân Thanh, Nguyen The Hung, Nguyen Thị Huyen Trang, Tran Ngọc Dien, Nguyen Thị Tuyen, Tran Xuan Truong, Tran Thị Dung, Nguyen Thi Phuong Thao, Vu Quang Lan

**Affiliations:** a School of Social Sciences Education, Vinh University, 182 Le Duan, Vinh, Nghe An, Viet Nam; b University of Science, Vietnam National University, Hanoi (VNU), 334, Nguyen Trai, Thanh Xuan, Hanoi, Viet Nam; c Vietnam Association of Geology, 6, Pham Ngu Lao, Hanoi, Viet Namw; d Institute of Geology, 84, Chua Lang, Dong Da, Hanoi, Viet Nam; e Vietnam Petroleum Institute, 167, Trung Kinh, Yen Hoa, Cau Giay, Hanoi, Viet Nam; f Hanoi University of Natural Resources and Environment, 41A, Phu Dien str. Hanoi, Viet Nam; g North Vietnam Geological Mapping Division, DGMV, Ai Mo, Gia Lam, Hanoi, Viet Nam

**Keywords:** Transgression, Regression, Systems tract, Lithofacies, Paleoshoreline, Deltaic lobe

## Abstract

The Red River Delta is considered one of the largest megadelta systems in Asia. The formation of this delta has been controlled by the continent-ocean interaction and sea-level fluctuation during the Cenozoic. In this study, we present a new sequence stratigraphic framework of the Red River Delta based on borehole lithofacies analysis and high resolution seismic data. The Late Pleistocene–Holocene sediments in the coastal zone of the Red River Delta were subdivided into three systems tracts: (1) the lowstand systems tract (LST) is characterized by a Late Pleistocene alluvial silty sand facies complex (arLSTQ_1_^3b^); (2) the transgressive systems tract (TST) is illustrated by the coastal marsh facies complex and the lagoonal greenish-gray clay facies of Early-Middle Holocene (amt, mtTSTQ_2_^1−2^); and (3) the highstand systems tract (HST) is composed of the Middle-Late Holocene deltaic clayish silt facies complex (amhHSTQ_2_^2−3^). The boundaries between these three systems tracts are not isochronous, namely: (1) The LST-HST boundary has been associated with the Würm 2 Glaciation, which occurred at ~40-18 Ka.; (2) The TST-LST boundary is identified by a transgressive erosion surface, whose age ranges from ~12-5 Ka.; and (3) the HST-TST boundary is an unconformity between the submarine deltaic facies complex and the Middle Holocene marine flooding plain.

## 1 Introduction

**Figure 1:**
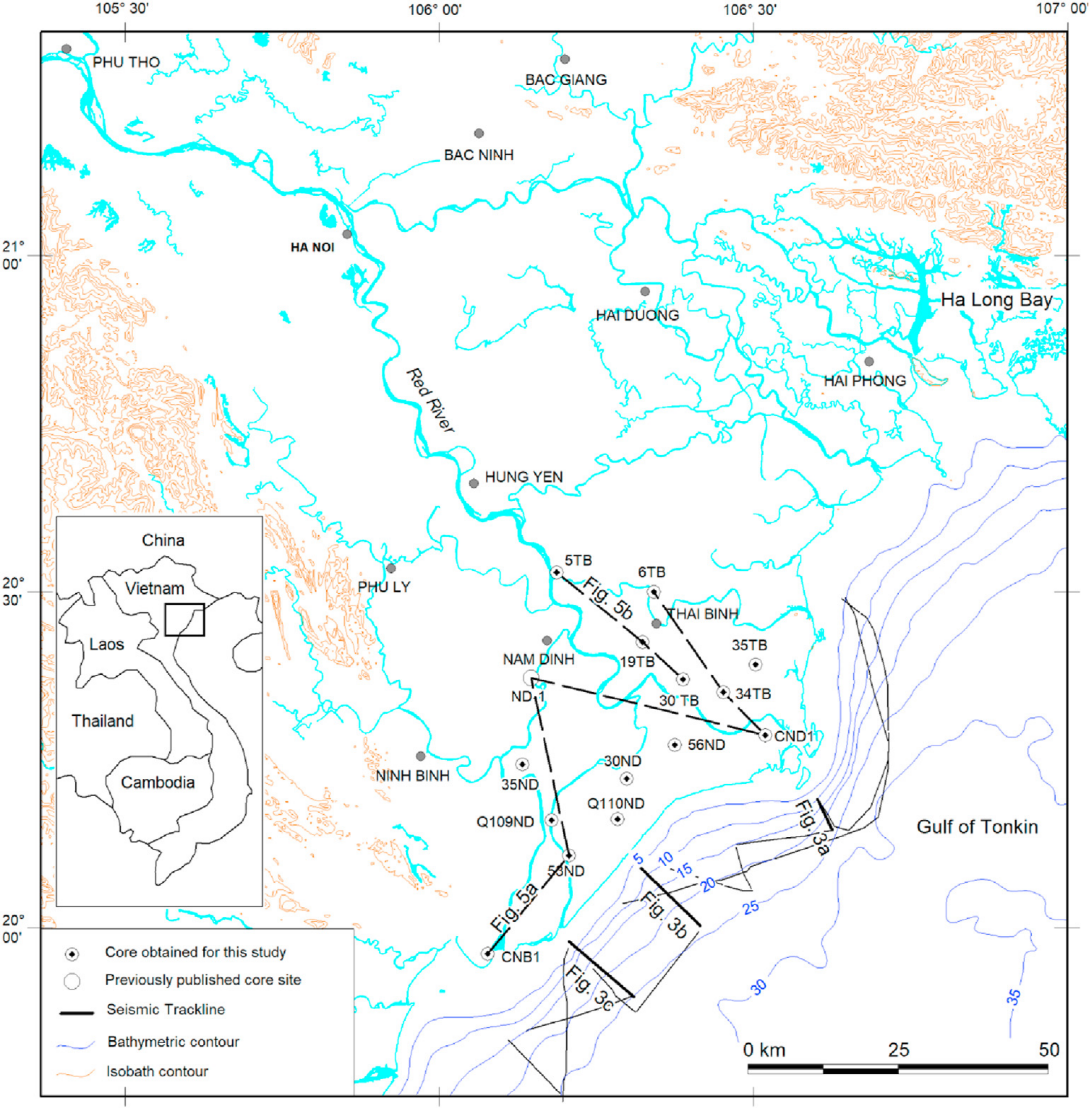
Location of the study area.

The Red River Delta extending over ~15,000 km^2^ is one of the largest delta systems around the world and the second largest delta in Vietnam after the Mekong River Delta in the South (Figure 1). The uppermost section of the delta consists of ~200m-thick unconsolidated overlying Tertiary weakly consolidated sedimentary rocks, which have been deposited during Red River pull-apart basin development in Cenozoic (Since ~34 Ma to present) (Nghi et al., 1991; Vietnam oil and gas Group, 2007). Formation of the delta – a part of a regional-scale Red River Pull-apart Basin was linked to the India-Eurasia collision and strike-slip motion along the Ailao Shan-Red River Shear Zone (Hall, 1998; HutChison, 2014; S. Zahirovic, M.Seton, 2014), which have been started since the Eocene. The Quaternary sediments of the Red River Delta have evolved for ~1.9 million years of development, which have been controlled by 5 cycles of global sea level change, corresponding to 5 cycles of glacial/interglacial events (Gunz/G-M; Mindel/R–W1; Wurm1/W1–W2; Wurm2/W2- Flandrian Transgression) (Nghi et al., 1991; Nghi and Toan, 1991; Tan et al., 2014).

The coastal zone of the Red River Delta is composed of: (1) the onshore land and (2) the nearshore area, extending from the Thai Binh River mouth in the North to the Day River mouth in the South (Figure 1). The onshore land is restricted from the modern shoreline to the end of the modern deltaic plain (at ~1,000 Ka. Shoreline position) while the submerged part is extended to a depth of ~30m of water depth, where is marked by the latest ancient shoreline associated with Flandrian transgression (~13000 Ka.).

Study of this area has been initially conducted together with geological mapping at 1:500.000, 1:200.000 and 1:50.000 scales. The obtained results of these projects allowed to subdivide the Quaternary sediments in this region into 5 formations: (1) Early Pleistocene Le Chi Formation (Q_1_^1^lc); (2) Middle-Late Pleistocene Ha Noi Formation (Q_1_^2−3a^ hn); (3) Late Pleistocene Vinh Phuc Formation (Q_1_^3b^vp); (4) Early-Middle Holocene Hai Hung Formation (Q_2_^1−2^hh), and (5) Late Holocene Thai Binh Formation (Q_2_^3^tb) (Khien, 2003; Ky, 1973; Thang, 1996; Toan, 1989).

Subsequent thematic researches have mainly focused on sedimentary cycles, geological evolution, material composition, and sedimentary facies (Lam, 2003; Lieu, 2006; Nghi, 2018; Nghi et al, 1991, 2002, 2016, 2017). One of the significant contributions from these studies is ^14^C absolute dating for the Holocene sediments (Ky, 1978; Lam, 2003; Mien and Phon, 2000; Nghi et al., 2002; Tanabe et al., 2003a) (Table 1). Numerous authors have attempted reconstruction of the morphological structure of the Holocene sediments and its linkage to the sea level change (Hanebuth et al., 2006; Li et al., 2006; Mathers and Zalasiewicz, 1999; Tanabe et al., 2003b).

**Table 1:** Results of the sedimentary age determination by ^14^C dating.

No	Sample note, Borehole	Location (coordinates)	Analysis material	Depth in m (compared to the Earth's surface)	Age (year BP)	Source	Place of Analysis—Labs
1	GA164844	20^o^15′26″ 106^o^30′57″	Animal crust	2.4	130 ± 40	Tanabe et al., (2006)	USA
2	ND-1	Vu Ban, ND 20^o^22′22″ 106^o^08′48″	Plant	3.2	505 ± 50	Funabiki et al., (2012)	Japan
3	GT-1	Giao Yen, Giao Thuy, ND 20^o^15′33″2 106^o^28′55″6	Shell, nail	0.5–1.0	560 ± 30	Doan Dinh Lam (2003)	Australia
4	LKCNĐ-1	Hillock 1 Giao Thuy	Plant	9.0	644 ± 23	Tran Nghi, Tran Thi Thanh Nhan, 2016	USA
6	NB164794	20^o^20′05″ 106^o^27′09″6	Wood	3.5	970 ± 40	Tanabe et al., (2006)	USA
7	CS-8	Tu Cac, Thai Hoa, Kien Xuong, TB	Plant	2.5–3.0	1340 ± 50	Doan Dinh Lam (2003)	ANSTO AMS, Sydney Australia
8	SC-2 OZF845	Binh Minh, Vu Thu, TB	Plant	0.4–0.5	1410 ± 40	Doan Dinh Lam (2003)	ANSTO AMS, Sydney Australia
9	CS-3	Le Loi, Kien Xuong,TB 20^o^26′37″7 106^o^27′38″7	Plant	2.8–3.0	1610 ± 4	Doan Dinh Lam (2003)	ANSTO AMS, Sydney Australia
10	HP336/3/2	Hoi Xuyen, Gia Loc, HD	Wood	2.0	4145 ± 50	Hoang Ngọc Ky (1978)	Germany
11	ND-1	Vu Ban, ND 20^o^22′22″ 106^o^08′48″	Plant	5.05	5280 ± 30	Funabiki et al., (2012)	Japan
12	VDC-25	Tam Coc, NB 20^o^13′42″ 105^o^55′47″	Shell	4.5–4.6	5300 ± 60	Doan Dinh Lam (2003)	Australia
13	HNK-7	Man Bac, Tam Diep, Ninh Binh	Animal crust	1.6	6860 ± 110	Nguyen Quang Mien, 2000	Vietnam Academy of Science and Technology (VAST)
14	MT-1	Me Tri, Thanh Xuan, HN	Peat	3.0	7100 ± 40	Tran Nghi (2017)	VAST
15	168815	20^o^41′05″ 106^o^08′48″	Wood	27.9	8490 ± 40	Tanabe et al., (2006)	USA
5	LKCNB-1	Thoi hillock Day mouth-NB	Shell, nail	21.0	9640 ± 30	Tran Nghi, Tran Thi Thanh Nhan, 2017	USA
16	HNK-34	Hong Thuan, Giao Thuy, ND	Wood	50.0	12,340 ± 115	Nguyen Quang Mien, 2000	VAST

Although the previous works brought remarkable contributions to the Quaternary sediment studies, there are still two main issues that have not been satisfactorily addressed:

(1) Firstly, in terms of stratigraphy, geological development, and sedimentary facies, the lithofacies and sequence stratigraphy framework have not been taken into consideration and therefore stratigraphic boundaries were not well matched. The Late Pleistocene-Holocene stratigraphy of the coastal zone was divided into 3 formations: The Late Pleistocene Vinh Phuc formation (Q_1_^3b^vp); The Early-Middle Holocene Hai Hung formation (Q_2_^1−2^ hh); The Late Holocene Thai Binh formation (Q_2_^3^tb). The boundary between Q_1_^3b^vp and Q_2_^1−2^hh was picked across the boundary between coastal marshy sediments and mottled clayish silt. Its age was determined as ~10 Ka. While the oldest mottled clayish silt has been recently dated at ~30 Ka. Based on ^14^C dating data; the boundary between Q_2_^1−2^hh and Q_2_^3^tb was delineated along the boundary between modern brown delta sediments (upper) and the underlying lagoonal greenish-gray clay layer, which was defined as 3 Ka.
(2) Secondly, studies on lithofacies-paleogeography, sedimentary evolutionary history, and hydrodynamic regime along the coastal area have long been considered to be more qualitative because the applied method is based on a combination of sedimentary physical properties and environmental geochemistry index. On the other hand, these works have not focused on the relationship between sedimentary evolution and global sea level changes in detail, meaning that it is not helpful for stratigraphical classification and construction of lithofacies - paleogeographic map, a very significant task in assessing the potential of the Quaternary groundwater aquifers.

In this study, the authors used a large number of sediment samples collected from 02 recently drilled boreholes and 12 archived cores distributed on the coastal land together with 25 shallow high resolution seismic profiles of the submerged part. In addition, we referred the interpretation of the Nam Dinh-1 core, which has been drilled by Tanabe et al., 2003a, Tanabe et al., 2003b. All of these data sets have been used and interpreted in order to reconstruct the evolutionary history of the Late Pleistocene - Holocene sediments sequence (Q_1_^3b^-Q_2_) and to provide a better understanding in distribution pattern and sediment evolution of the study area.

## 2 Materials and methodology

### 2.1 Materials

Apart from a large number of previously published data, samples, and logging data collected from 14 coastal boreholes have been subjected to different analyses and interpretation purposes. The location of these boreholes is displayed in Figure 1. Core samples have been used for ^14^C dating and sedimentary structure study. These boreholes have been drilled at different points of time, the previous work simply logged the core under naked eye description without any detail lithofacies or physical property logging of the sediments were given. In this study, we have done more detailed interpretation based on lithofacies together with sedimentary physical properties analysis, ^14^C dating, and high-resolution seismic interpretation. This re-interpretation allowed us to define the sedimentation and depositional environments of the Red River Delta and its linkage to the sea-level change during the Holocene much clearer.

Besides, 25 high resolution seismic lines off the Red River Delta were used for seismic and sequence stratigraphy interpretation (Figure 1 and Table 1).

### 2.2 Methodology

#### 2.2.1 Accommodation space and sequence stratigraphy during the transgression and regression

Sequence stratigraphy has been widely applied around the world since the 1970s, last Century. However, the proper application depends on the particular condition of regional tectonics and the depositional system in each area. Studying Quaternary lithofacies and sedimentary environment of coastal plains and nearshore areas in Vietnam have been carried out by a number of geoscientists such as Tran et al. (Nghi et al, 2002, 2003). These works have established a connection between the space of sedimentary accumulation and transgressive and regressive phases; In principle, these are the same and extended from an eroded surface and correlative conformity surface to the center of the sedimentary basins. From this standpoint, the sediments often accumulate in that space, but the only spatial difference is the facies and environment. following the transgressive and regressive direction, the lithofacies would continuously change over space and time.

#### 2.2.2 Selection of sedimentary cycle boundary

**Figure 2:**
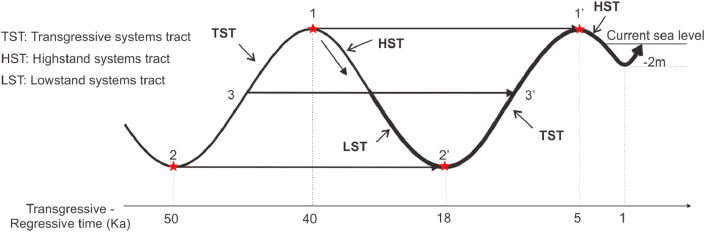
The boundary of the sedimentary cycle taken as line (33′) which coincided with the sequence boundary (Modified from Nghi T., 2012).

There are three ways to pick sedimentary cycle boundaries in this study (Figure 2): (1) Selection of the boundary at the maximum transgressive location (11′); (2) Selection of the boundary at the minimum regressive location (22′); And picking boundary at the average sea level (33’). Nghi (2018) selected line 33” based on the following reasons: (1) This boundary coincides with the eroded surfaces of the channels, and hence they can be easily recognized on cross-sections and/or borehole logs; and (2) This boundary is also coincident with the boundaries of sequences defined in the sequence stratigraphy, i.e., unconformities of channels generated by regressive phases (Nghi, 2018).

Each sedimentary cycle corresponds to each sequence containing three systems tracts: (1) the alluvial facies complex corresponding to the lowstand systems tract (arLST); (2) the coastal, shallow marine, and lagoonal facies complexes equivalent to the transgressive systems tract (amt, mtTST); and (3) the deltaic facies complex comparable to the highstand systems tract (amhHST).

Our study on the evolution of the Late Pleistocene–Holocene sediments in the coastal zone of the Red River Delta was based on two important lithofacies analysis methods: (1) Analysis of lithofacies and the sedimentary environment based on lithological parameters and (2) Lithofacies analysis based on interpretation of high-resolution seismic profiles following standards of the sequence stratigraphy.

#### 2.2.3 Lithofacies analysis based on indicative sedimentary physical properties

Lithofacies analysis is an integration of sedimentary and geochemical proxies indicating the sedimentary environment in order to delineate the same age lithofacies units according to space and time. The workflow of lithofacies analysis is carried out in 2 steps: (1) Analysis of indicative sedimentary physical properties, which reflect sediment dynamics and deposition environment; (2) Detail interpretation of lithofacies and sedimentary environment based on the former parameters.

(1) Analysis and calculation of indicative environmental parameters:
a) *Granulometric properties include* mean (Md), sortness (So) values, which were derived from statistic computation of the grain size distribution from 300 samples taken in 14 boreholes proposed by Trask, P.D (1932) (Trask, 1932).
Md (mm) is the average size of the particle. Md reflects the river hydrodynamic regime, transportation distance, and nearshore wave dynamics.
So - the sorting coefficient is a very important property in lithofacies analysis. According to Rukhin (1969), So = 1–1.58 demonstrates good sorting, typical for tidal flat sand facies with strong active waves, river mouth sand bar facies and sandy barrier bar; So = 1.58–2.12 corresponds to medium sorting, typical for downstream river channel, deltaic plain environment while So > 2.12 characterizes flood plain, delta plains and mixed tidal flat environments (Rukhin, 1969).
b) *Particle roundness coefficient (Ro):* Roundness coefficient (Ro) is a property for the morphology of sediment grains reflecting abrasion on the particle during its migration. The values of Ro were deduced from 100 sandy thin sections taken from 14 boreholes. The roundness coefficient is an index representing the degree of rounding of the grains during the transportation and deposition process. Ro is calculated by a formula as follow (Nghi, 2003):

Ro = 1–0.1n

Where n is the number of convex angles of the particle. Ro varies from 0 (min) to 1 (max), implying transportation distance of the clastic sediments from the sources and is of great significance in lithofacies analysis and sedimentary environment interpretation. Ro = 0.0–0.3 indicates the deluvial and proluvial environments, Ro = 0.3–0.5 indicates the river bed environment, Ro = 0.5–0.7 suggests a deltaic, tidal flat, and river channel sand bar environment while Ro > 0.7 is an indication of the river mouth sandy bar and tidal flat with strong wave activity (Nghi, 2003).

c) *Q (%)* is the quartz index varying from 0 (min) to 1 (max), which were estimated from 100 thin sections of unconsolidated sands taken from 14 boreholes. The method applied for coalesced unconsolidated sediments, which was proposed by Tran Et al, 2003 (Nghi, 2003).
d) *Acid-alkali index (pH)* was analyzed for 50 clay samples taken from 14 boreholes: pH = -lg H ^+^. If pH < 7, it is interpreted as the continental environment; pH = 7 means neutral environment (transition between continental and marine environment) while marine environment shows pH > 7.
e) *Redox potential (Eh) (mV)* was analyzed for 50 clay samples taken from 114 boreholes. In nature, it varies from -200mV to +500mV. When Eh < 0, sediment is likely associated with a coastal swamp environment.
f) *Exchange cation index* was analyzed for 50 clay samples taken from 14 boreholes: $Kt=\frac{K^{+}+Na^{+}}{Ca^{+2}+Mg^{+2}}$ is calculated in mgd/100g (milliequivalents of charge per 100 g of dry soil). Kt < 0.5 is typical value for continental environment; Kt = 0.5–1.0 shows transition environment while Kt > 1 is attributed to marine environment.
(2). Comprehensive interpretation of lithofacies based on sedimentary and geochemistry data:

Each sedimentary facies are characterized by two components: (1) sedimentary composition and (2) depositional environment. In order to determine the name of a certain lithofacies from any sediment sample, it is basically dependent on lithological parameters and indicative environmental geochemistry. For example, a sandy sample owning So = 1.3; Ro = 0.7; Q = 0.95 is attributed to belong to a wave-dominated tidal flat sand facies while a black mud sample of coastal swamp mud facies commonly shows So = 3.0; pH = 4.5; Eh = -10 mV and Kt = 1.8. Specific physical properties and indicative geochemical indexes for each typical facies and environment are presented in Table 2 and the lithofacies-paleogeographical maps of the coastal zone of the Red River Delta were eventually established.

**Table 2:** Integration of typical sediment parameters for coastal zone lithofacies of the Red River Delta. Note: Q, Quartz content; So, Sorting coefficient; Ro, Roundness coefficient; pH, Acid-alkali index; Eh, oxidation-reduction potential index (mv); Kt, Exchange cation index (mgd/100 g sample); S, Ratio of sand to mud.

TT	Parameters							
	Lithofacies	Q (%)	So	Ro	S	pH	Eh (mv)	Kt
1	River channel sand facies	45–60	2.3–3.0	0.3–0.5	8/2–10/0			
2	River mouth sand bar facies	80–95	1.2–1.5	0.6–0.8	7/3–10/0			
3	Flood plain clayey silt facies		2.5–3.5			6.2–6.7	100–200	0.1–0.6
4	Coastal swamp mud facies		2.5–3.5		1/9–3/7	4.5–6.0	−15 to −50	0.9–1.2
5	River mouth lagoon mud facie				1/9–3/7	7.5–8.2	−10 to +50	1.0–1.5
6	Marine flooding plain clay facies		1.7–2.4		1/9–2/8	8.0–8.5	10–100	1.5–2.0
7	Delta front sandy mud facies		1.3–2.9		2/8–4/6	7.5–8.0	20–100	1.2–1.8
8	Prodelta clay facies		1.6–2.5		0/10–2/8	8.2–8.6	20–100	1.8–2.3

#### 2.2.4 Interpretation of high-resolution seismic data sections and sequence stratigraphy

**Figure 3:**
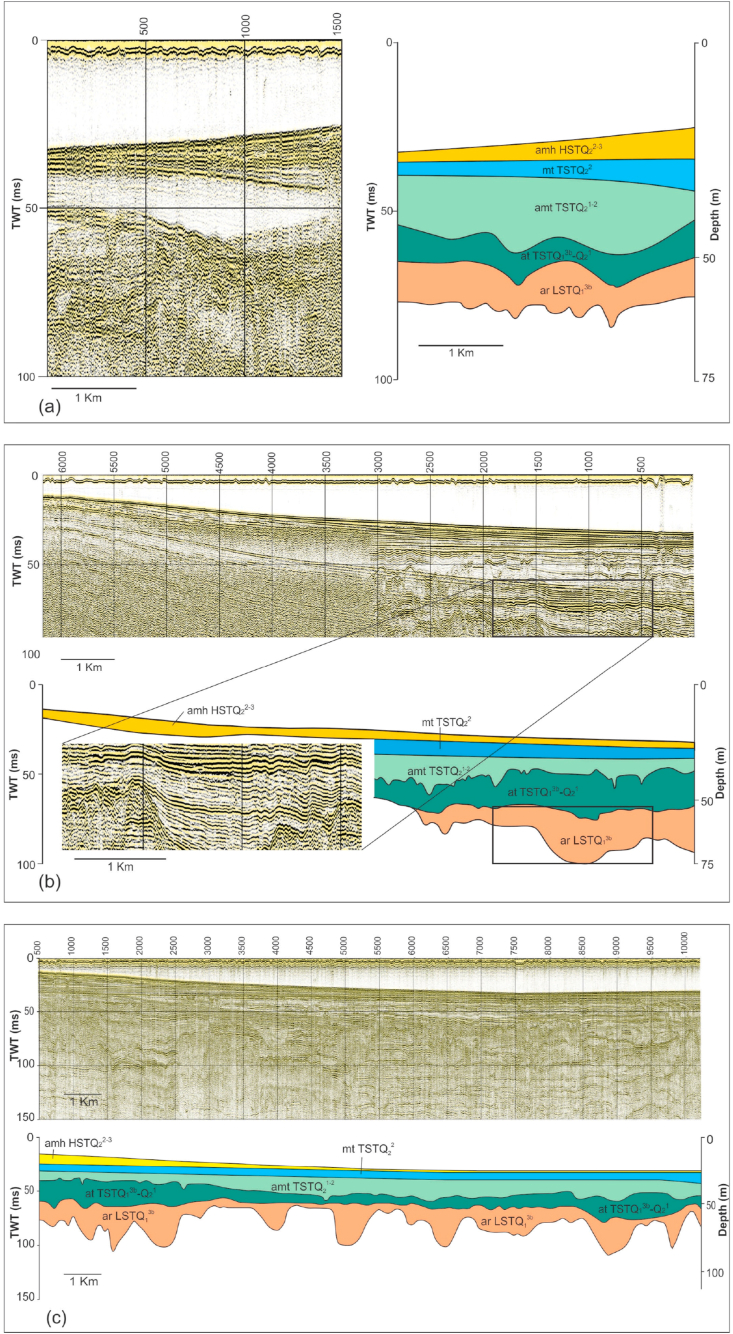
Interpreted high-resolution shallow seismic sections: (a) line T14-CH1 in the Ba Lat mouth area; (b) line T6-CH1 in the Hai Hau area; (c) line T22-CH1 in the Ninh Co mouth area (section locations are shown in Figure 1, legend in Figure 5).

Our seismic interpretation showed that there is a close relation between seismic facies/reflection configuration and sedimentary depositional systems tracts. This linkage is described as follows (Figure 3):

- Lowstand systems tract (arLSTQ_1_^3b^) is characterized by strong to medium amplitude, progradation seismic reflection configuration, which is characterized by the parallel reflection of seismic signals in the upper part and chaotic configuration in the lower part, low frequency represents for the sand bodies in the incised valleys of regressive channels and the alluvial clayish silt facies. The lower boundary is marked by an incised surface of the river channel.
- Transgressive systems tract (at, amt, mtTSTQ_2_^1−2^) can be identified by onlapping seismic termination in the basin margin, low amplitude, discontinuity, high frequency, and parallel configuration of seismic reflection, indicating coastal and lagoonal environments. The lower boundary is determined by a transgressive erosion surface.
- Highstand systems tract (amhHSTQ_2_^3^) can be recognized by an alternative succession of high/low amplitude, parallel pattern, and a downlapping termination of seismic reflections (progressive wedge structure of the submarine delta), showing the successive coarse/fine grained sedimentary packages. The lower boundary is picked across the downlapping termination surface, which is underlain by the marine flooding plain.

## 3 Results and discussion

### 3.1 Lithofacies analysis

#### 3.1.1 Lithofacies of the Late Pleistocene Lowstand Systems Tract (LSTQ_1_^3b^)

**Figure 4:**
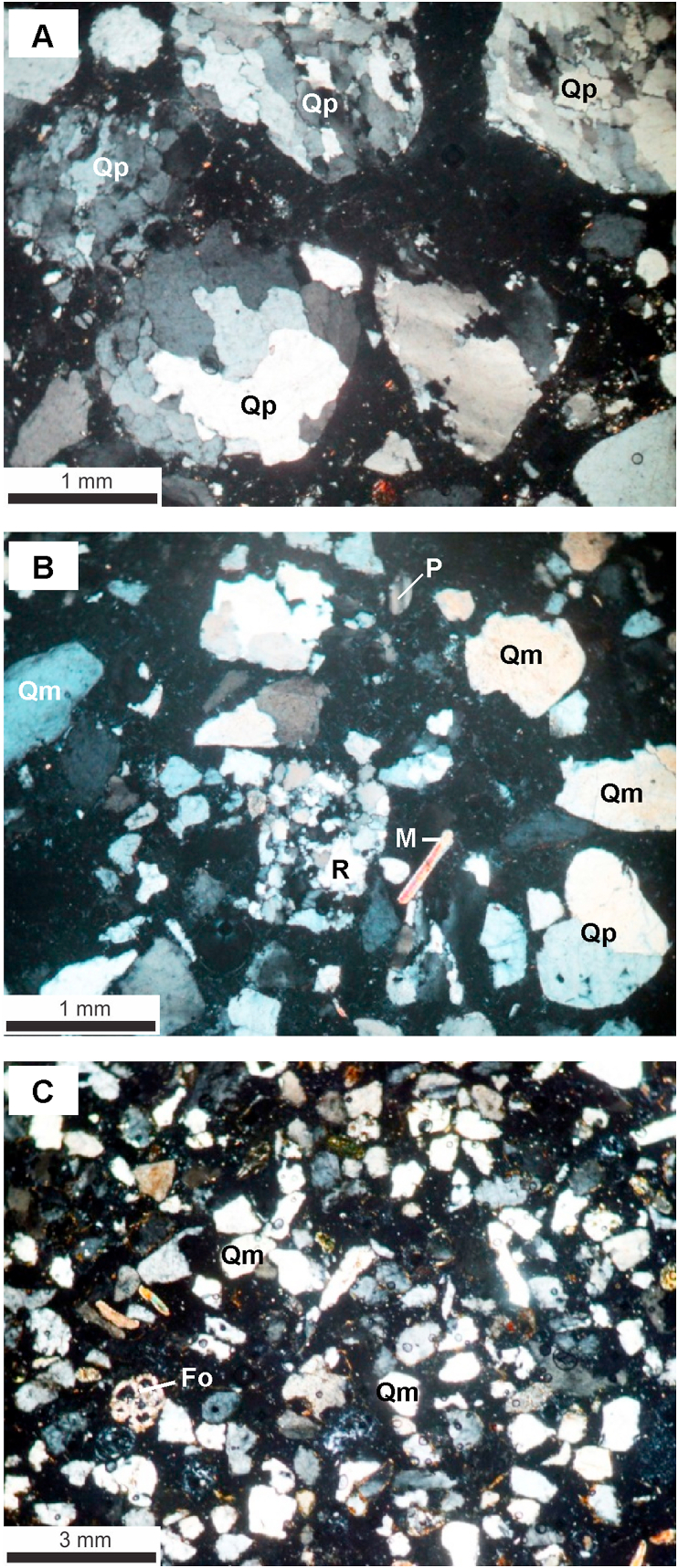
Photos of thin sections of fine sand under a polarizing microscope, from borehole 56 - ND-1a, N^+^. a. River channel polymictic sand with medium grain size in the lowstand, poor sortness, and average roundness. Rich in Quartz (Qp) belonging to the lowstand systems tract, age Late Pleistocene (arLSTQ_1_^3b^); b. River channel polymictic sand with small grain size in the transgressive systems tract, age Late Pleistocene–early Holocene (atTSTQ_1_^3b^-Q_2_^1^), and poor sortness and roundness; c. Tidal flat oligomictic sand in the transgressive systems tract with age early Holocene (amtTSTQ_2_^1^). Relatively good sortness and roundness and rich in biological fragments, mica, and laterite grains. Note: Qm, monocrystal quartz; Qp, polycrystal quartz; M, muscovite; P, plagioclase; R, fragment of rock; Fo, fragment of foraminifera.

Within coastal zone of the Red River Delta, the Late Pleistocene Lowstand Systems Tract (Q_1_^3b^) is mainly composed of regressive alluvial facies complex (ar LSTQ_1_^3b^) and was observed at ~5–14 m deep (Table 3). On high resolution seismic profiles, this facies complex directly overlies the channel-eroded regressive surface (Figure 3). Our core description showed that this complex is composed of two facies: (1) The lower facies is a fluvial channel, polymineral sand facies with poor sortness and roundness (So = 2.5; Ro = 0.4) (Figures 4 and 6) and (2) the floodplain silty clay of the upper facies is characterized by poor sortness (Figure 6).

**Table 3:** A summary table of the depths and thicknesses (m) of the boundaries of lithofacies and systems tracts of the Late Pleistocene-Holocene in the Ba Lat river mouth area.

System Tract	Age	Lithofacies	30TB		6TB		35BT		5TB		34BT		19TB		56ND		35ND		Q110ND		Q109ND		53ND		30ND		CND1		CNB1		ND-1	
			T	cD	T	cD	T	cD	T	cD	T	cD	T	cD	T	cD	T	cD	T	cD	T	cD	T	cD	T	cD	T	cD	T	cD	T	cD
HST	Q_2_^3^	amh HST	13	13	2	2	11	11	11	11	12	12	14	14	19	19	9	9	10	10	11	11	14.7	14.7	14	14	12	12	20	20	9	9
TST	Q_2_^1−2^	mt TST	5	18	6	8	13	24	6	17	12	24	6	20	6	25	21	30	5	15	9	20	3.8	18.5	8	22	8	20	2	22	10	19
		amt TST	12	30	10	18	8	32	10	27	8	32	14	34	14	39	24	54	7	22	12	32	4.5	23	8	30	22	42	11	33	35	54
		at TST	10	40	11	29	5.5	37.5	9	36	9	41	10	44	8	47	16	70	8	30	8	40	6	29	9.5	39.5	-	-	-	-	10	64
LST	Q_1_^3b^	ar LST	14	54	-	-	9.5	47	11	47	11.5	52.5	11	55	9	56	-	-	6.6	36.6	6	46	7	36	6.5	46	-	-	-	-	6	70

cD: Cumulative depth of facies group (m).

T: Thickness of facies group (m).

Seismic facies of the Q_1_^3b^-Q_2_ deposits are relatively high frequency and the weakly erosional surface of the Day paleo-River (A tributary of the red River system), which were observed in some place.

#### 3.1.2 Lithofacies of the end of the middle pliocene - Late Pleistocene transgressive systems tract (TST Q_1_^3b^-Q_2_^2^)

In the lower part of the Red River Delta, the Transgressive Systems Tract is ~20–30m thick and was subdivided into three basic facies groups as follows (Table 3):

*The Late Pleistocene - Early Holocene transgressive alluvial facies complex (atTSTQ*_*1*_^*3b*^*-Q*_*2*_^*1*^*):* This complex directly overlies the regressive alluvial facies group (arQ_1_^3b^) and is identified by a combination of lithofacies analysis and sequence stratigraphy interpretation. Formation of this special facies Complex can be explained as follows: When the Flandrian transgression started, the coastline was still located far away from the present shoreline position; Due to sediment supply dominated over accommodation space creation, the Red River Delta has continued prograded to produce such an alluvial facies complex. This is known as the transgressive alluvial complex, belonging to the transgressive systems tract (atTST). Results of thin sections examined for this facies complex showed that the sediment composition is of polymineral, distinctive sortness, and roundness values for the fluvial channel tributaries of the deltaic plain (Figure 4b).

*The Early Holocene Transgressive coastal facies complex (amtTSTQ*_*2*_^*1*^*):* It consists of three facies: tidal flat sand, estuary sandy mud and coastal lagoon clay and swamp peat facies. This facies complex is situated from ~47 - 24 m deep and the age varies from ~10-8 Ka., creating a wide paleoshoreline zone over ~5–7 km long and is coincident with the present shoreline zone (Figure 7b).

- The tidal flat sand facies is directly underlain by a transgressive erosion surface, whose age varies from ~10 - 8 Ka. and is distributed at ~ 47-24 m deep (Table 3). The facies are composed of medium/fined grained sand, containing lateritic gravelly sand and shall fragments with roundness Ro = 0.5–0.7 and average sortness So = 1.8. Origin of these lateritic materials have been likely derived from chemical weathering product during the exposition of the paleo-continental shelf, which is commonly seen in the Early Late Pleistocene (Q_1_^3a^) mottled silty clay layers.
- The estuary sandy mud facies accounts for >50% mud content, pH = 7.0–7.5, and poor sortness (So > 3.5) (Figure 6).
- The lagoonal clay and swamp peat facies demonstrate >30% clay content, total organic carbon (TOC) = 5%–20%, pH = 4–7.5, Eh < 0, and bad sortness (So > 3.0).

*The lagoonal greenish gray clay facies group (mtTSTQ*_*2*_^*2*^*)* was restricted within the ~6 - 5 Ka. maximum transgressive paleoshoreline zone (Figure 7c). The lagoonal greenish gray clay facies are distributed at a depth of ~7–25 m with an average thickness of ~8m (Table 3). Typical characteristics of this facies are very high clay content (>70%), where montmorillonite accounts for >20% of the total clay minerals, pH = 7.5–8.0 (Figure 6).

#### 3.1.3 Lithofacies of the Middle-Late Holocene highstand systems tract (from 5 Ka. - Present) (amhHSTQ_2_^2−3^)

The highstand systems tract (HST) started from ~5 Ka. and is characterized by the deltaic facies complex, which has constituted the recent Red River Delta. Evidence of the paleo-shoreline was identified by 1 – 3m (up to 5m) high sea notches situated in limestone cliffs in Ninh Binh province, the margin of the paleo-Red River Delta. Our ^14^C dating of shellfish fragments collected from these sea notches gave their age of ~5 Ka. (Lam, 2003, Table 1). In the coastal zone of the Red River Delta, four ancient shoreline generations can be identified based on the ages given by ^14^C dating of shellfish (Lam, 2003, Table 1) as follows: ~3–2.5 Ka., ~1.5–1 Ka., ~0.7–0.5 Ka. and ~0.2–0.15 Ka. (Figure 7d). Each ancient shoreline generation was associated with a "flower bunch”-like of the paleo-deltaic lobe (Figure 7).

The highstand systems tract is composed of three lithofacies complexes: (a) the Middle-Late Holocene burial submarine deltaic facies complex, (amhHSTQ_2_^2−3^); (b) the delta plain facies complex, including deltaic flood plain clayish silt facies and sand ridge facies distributed on the plain surface; and (c) the modern submarine deltaic facies complexes (amhHSTQ_2_^3^) is distributed from 0 to 20m deep. This complex consists of delta front sandy mud facies and prodelta clay facies.

**Figure 5:**
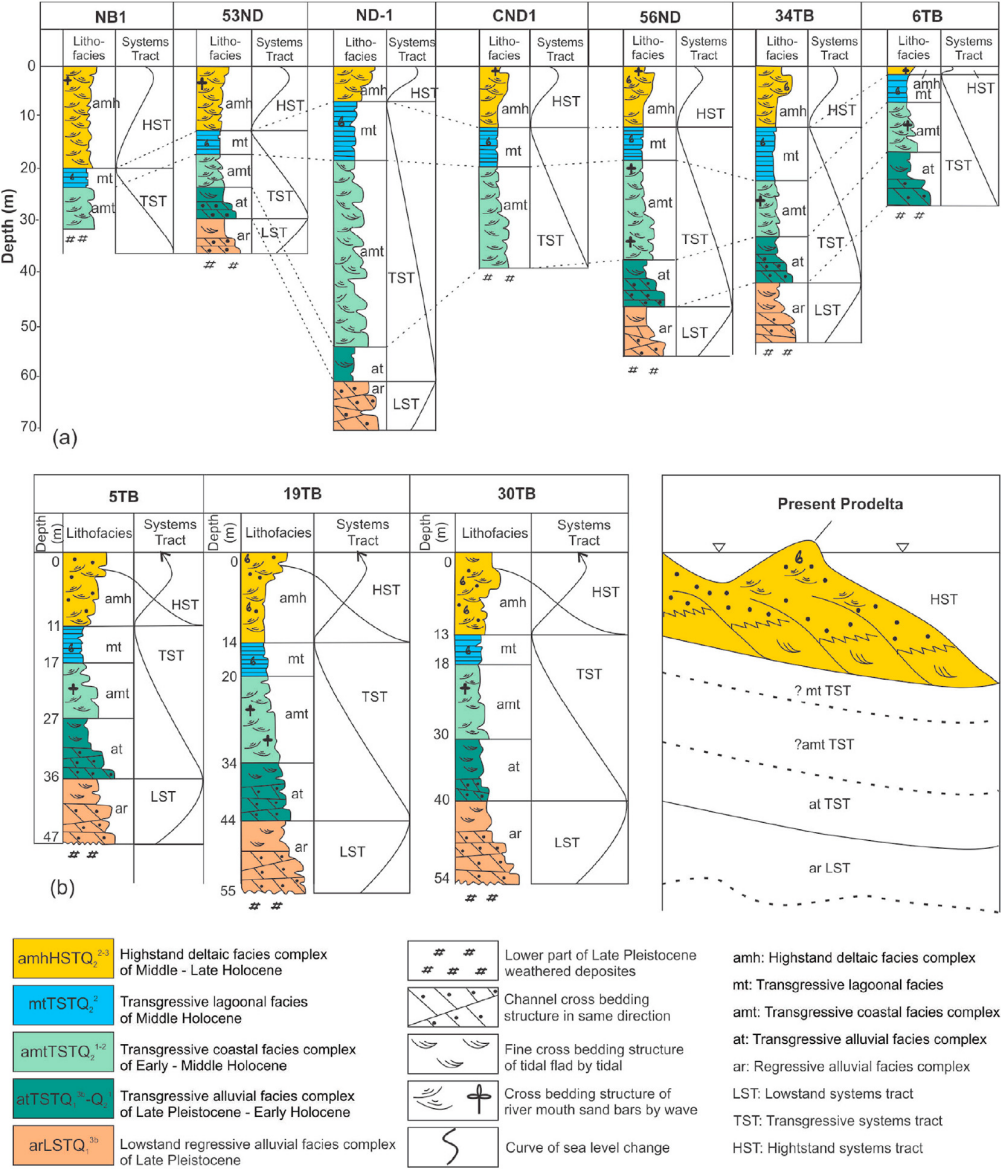
Sediment core logs and depositional facies. Core locations are shown in Figure 1.

**Figure 6:**
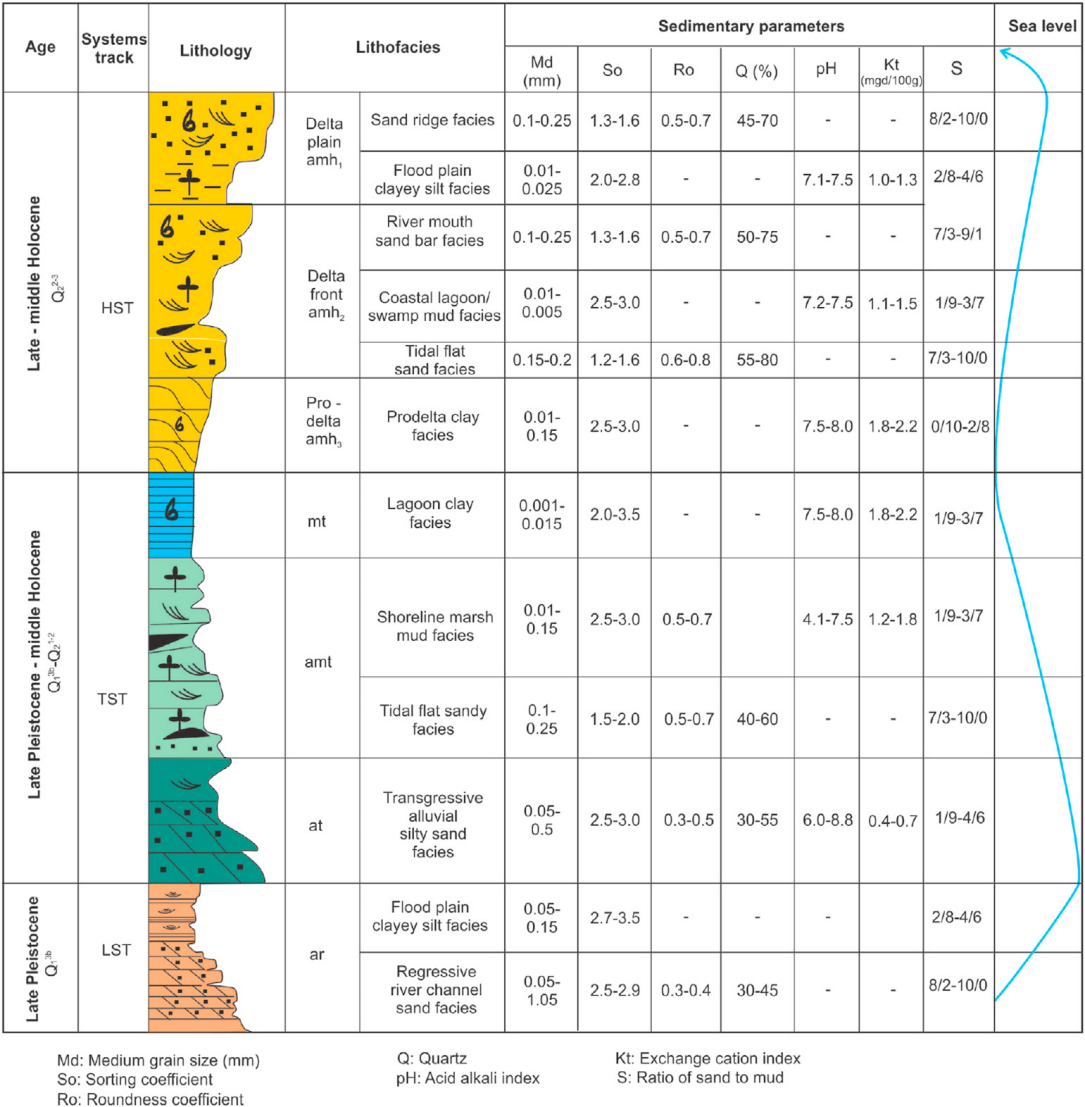
Summary of facies and sedimentary parameters by systems tracts (see legend in Figure 5).

**Figure 7:**
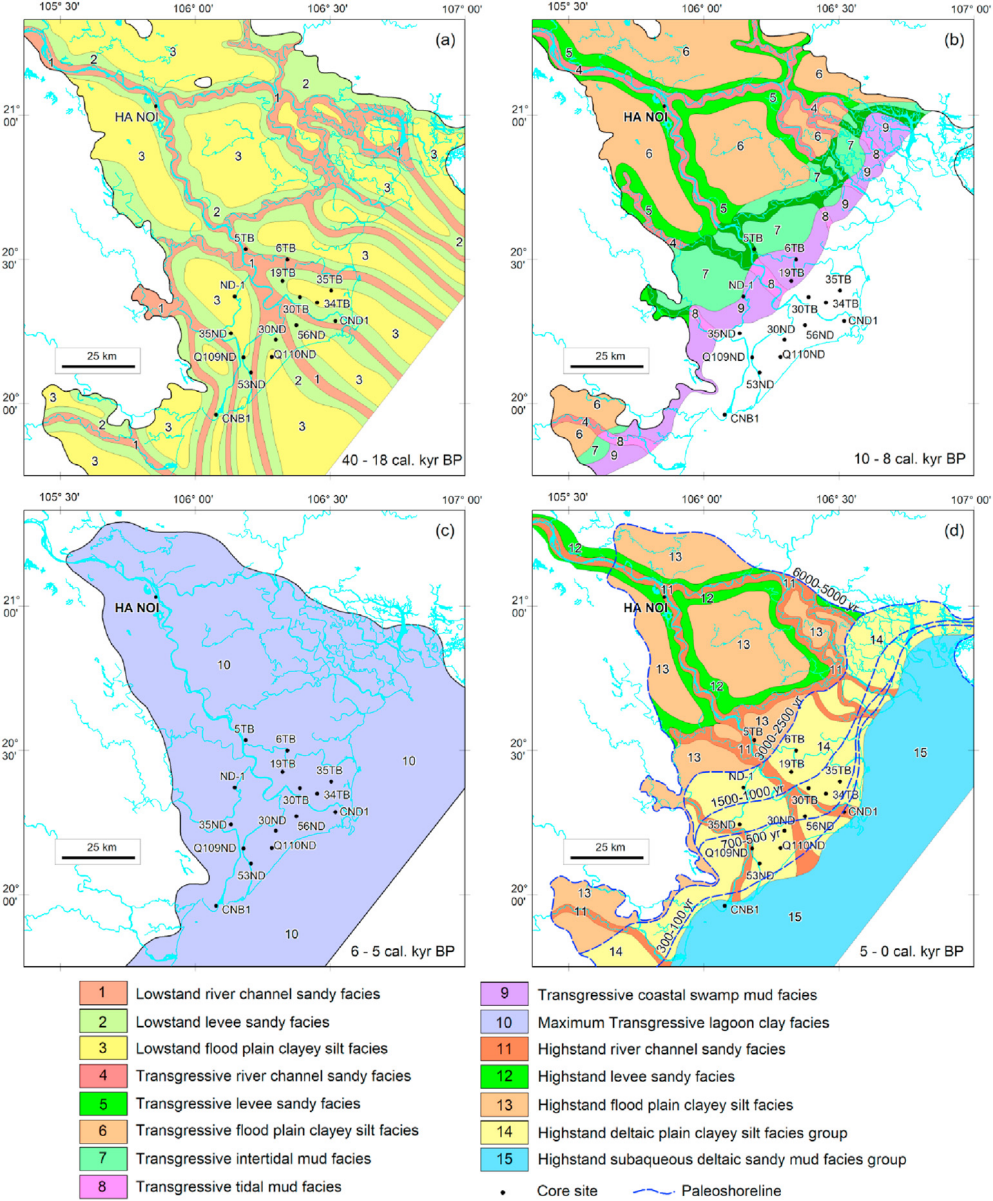
Paleogeographic maps for the different stages from Late Pleistocene to late-middle Holocene. a) The stage of the Late Pleistocene which belongs to the lowstand systems tract. The alluvial facies complex dominates (arLSTQ_1_^3b^); b) In the early Holocene stage, the sea level was interrupted for 2000 years (10-8 Ka), forming a paleoshoreline zone which nearly coincided with the present one and which belonged to the transgressive systems tract with two lithofacies complexes: the transgressive alluvial lithofacies complex and the coastal swamp lithofacies complex (at, amt TSTQ_2_^1^); c) The middle Holocene maximum transgressive stage with interrupted sea level for 1000 years (6-5 Ka) formed the paleoshoreline marked by the marine notch in the limestone cliff at the Ninh Binh province and the marine terrace 5 m high running along the marginal area of the Red River delta plain; d) The middle-late Holocene regressive stage created the alluvial delta plain marked by four paleoshorelines with ages of 3–2.5 Ka: 1.5–1 Ka, 0.7–0.5 Ka, and 0.3–0.1 Ka belonging to the highstand systems tract (amhHSTQ_2_^2−3^).

**Distribution of lithofacies complexes** (Figures 5, 6, and 7):

a. The *Middle-Late Holocene submarine deltaic facies complex (amh*_*3-2*_*HSTQ*_*2*_^*2−3*^*)*

This lithofacies complex lies at a depth of ~2–20m due to modern tectonic subsidence and/or sea-level rise (Figure 6) and includes two lithofacies: (1) The prodelta clay facies (lower part) formed a progressive wedge-shaped structure that extends from the mainland to the sea which overlies the Middle Holocene marine flooding plain clay facies (mt TSTQ _2_^2^); (2) the delta front sandy mud facies overlying the prodelta clay facies is characterized by the horizontal bedding structure which has been controlled by the coastal bottom current dynamics.

b. *The Late Holocene delta plain facies complex (amh*_*1*_*HSTQ*_*2*_^*3*^*)*

This lithofacies complex is extended from ~2.5 Ka.-age shoreline to the modern shoreline position (Figure 7d). Based on the plain elevation relative to the modern sea level and lithological parameters, we can divide the delta plain into two groups of lithofacies: (1) the upper deltaic freshwater-bearing clayish silt facies group and (2) the lower deltaic brackish-water-bearing silty clay facies group.

*The upper deltaic freshwater-bearing clayish silt facies group*

This facies group is distributed from ~2.5 Ka.-age shoreline to ~1.0 Ka.-age shoreline and has deposited in the freshwater environment (pH = 6.5–7.2). This group is illustrated by three typical features:

- Sand ridge facies

Sand ridges are crescent shaped, and asymmetrical bow heading to the sea. They commonly extend over a width of ~1000–5000m, a height of ~3–7m, and a length of ~5000 - 20,000m. They are formed in the front of the ancient river mouths within wave-breaking zones. Each generation of sand ridges marks a shoreline position during the regression process in the Late Holocene. They have retrograded gradually and are younger from the mainland to the sea (Figure 7d). The sand ridges are dominated by sand fraction (>60%), medium to good roundness, and sortness of detrital grains (Ro = 0.4–0.7; So = 1.3–1.9) (Figure 6).

- Flood delta plain clayish silt facies

The flood delta plain clayish silt facies are distributed between sand ridges, whose height varies from ~2 to 3m above modern sea level. They are not inundated by the sea water during the spring tide season. This lithofacies is characterized by fresh-water containment, high clay fraction (accounting for >70%), poor sortness (So > 3.5), and pH varying from 6.9 to 7.2 (Figure 6).

*The lower deltaic brackish-water-bearing silty clay facies group*
This lithofacies is distributed from ~1.0 Ka.-age to the modern shoreline and is distinguished by a brackish water environment (pH = 7.2–7.5). Elevation of the plain surface ranges between 2 to 0 m above present day sea level (Figure 7d) and is subdivided into five facies:
- The flood deltaic clayish silt clay facies containing >75% clay with poor sortness (So > 3).
- The lower deltaic sand ridge facies. Similar to the upper delta, the sand ridges in the lower delta also have a bow and crescent shape turning to the sea and divided into two branches at the southern tail due to the ancient longshore current flowing southward.
- Coastal swamp mud facies of the lower delta. The lower delta plain is a muddy tidal flat that is flooded by the sea during the high tide condition. Composition of sediments is mainly silty clay with poor sortness (So = 3.5), pH = 7.2–7.8, and Kt = 1.2–1.5. Brackish water trees and mangrove swamp are presently well grown in this area.
- Distributary and tidal channel mud facies of the lower delta plain. Distributaries and the tidal channel have been formed simultaneously with the accretion process of the lower delta from the mainland to the sea. During the dry season, they were the distributed flows of the Red River. During storm surges, the channels became submerged under sea water. At that time, sedimentation process was controlled by a mixing of the riverine-marine hydrodynamic regimes.
c *The modern submarine lithofacies complex (amhHSTQ*_*2*_^*3*^*)*
Boundary between the delta plain and front delta has been defined by the authors of this article as being the waterline at the highest tide (Nghi, 2018; Nghi et al, 2016, 2017). According to this classification scheme, the submarine delta facies complex of the Red River can be splitted into two basic facies groups: (1) the delta front facies group and (2) the prodelta facies group.
*Delta front facies group composed of four lithofacies:*
- The modern tidal flat mud facies and the intertidal mud facies are distributed between the highest and the lowest tide level. The composition of sediments are mainly mud (>70%), with TOC ranging from ~2% to 10% and containing peat in some places. The depositional environment representing these facies is anoxic and dominant acidic regimes (Eh ≤ 0, pH ≤ 4), which are indicated by the presence of organic matter and the formation of pyrite minerals during the sedimentation.
- Modern river mouth lagoon mud facies: The modern river mouth lagoon mud facies of the Ba Lat river mouth is separated from the sea by the Vanh, and Thoi Sand Bars, creating a semi-enclosed water body connected to the sea in three directions, via the river mouth and two tidal channels that flow into the sea in the northeast and southeast. The estuarine lagoon becomes narrower due to the "convergence" of different sedimentation processes. Association with this environment is the semi-enclosed lagoonal mud facies, where mangrove swamps are growing, similar to the marshy mud flats along the tidal coast.
- Modern sand bar facies: The modern sand bars of the Red River Delta include the Den, Vanh, Lu, Ngan, Thoi, and Mo Sand bars. These sand bar systems mark the 5^th^ generation of the shoreline since the last ~2.5 Ka. compared to the previous ancient sand bars. It is demonstrated by a high concentration of quartz (Q reaches over 90%), very poor sortness (So: 1.2–1.3), and good roundness (Ro: 0.5–0.9).
- Coastal shallow water sandy mud facies: The coastal shallow water sandy mud facies are distributed from a depth of 0m–15m, creating a submarine, flat, fan-shaped plain, tilting seaward at an angle of ~0.5-1^o^. Characteristics of the sediment properties are poor sortness (So: 2.3–2.7), relatively low sand/mud ratio (1/9 - 4/6), and quite high acid-alkaline index (pH: 7.2–8.5).

The prodelta clay facies group: The prodelta clay facies group is distributed from ~15 - 20m deep, tilted at ~5^o^-15^o^ at the slope of the submarine delta. This is the distal limit of the submarine Red River Delta. The foot of the prodelta become gradually thinner toward the modern sea. This is a mud facies distributed on flat terrain and is belonged to the highstand systems tract (amhHSTQ_2_^3^).

### 3.2 Sedimentary evolution and cross-stratigraphical boundaries

#### 3.2.1 The Late Pleistocene regressive stage is correlative to the lowstand systems tract (Q_1_^3b^ LST), which occurred from 40 to 18 Ka

During this period of time, the whole Red River delta had been exposed. Two exogenous geological processes took place: (1) Sediment deposition to produce an alluvial sedimentary rhythm, including two facies distributed from the bottom to the top as follow: a) polymictic channel sand facies with poor sortness and roundness, directly overlies the eroded surface of the river channel and b) flood plain clayish silt facies. (2) Beginning of the Late Pleistocene (Q_1_^3a^) weathering process occurred on the deltaic clayish silt deposits of the Vinh Phuc Formation. this chemical weathering has resulted in the formation of a laterite crust above the deltaic clayish silt layer that was identified by mottled texture and was considered the marker layer. The chemically weathered/eroded surface was widely observed at different depths from onshore to a water depth of ~100m on the Vietnam continental shelf, producing a cross-boundary in the stratigraphy of the region, whose age gradually becomes younger from the onshore to the offshore (40 - 18 Ka.).

#### 3.2.2 *The Late Pleistocene-Middle Holocene (~18-5 Ka.) Flandrian transgression*, corresponding to the transgressive systems tract (Q_1_^3b -^ Q_2_^2^ TST). This systems tract is composed of three facies groups as follow

- The *Transgressive alluvial facies group (at)* started in the transgressive phase and is situated at a depth of 100 m, where is coeval with the paleo-shoreline of the Würm 2 Glaciation (18 Ka.) and the rate of sea level rise at that time was estimated at ~5 mm/year (Nghi, 2018). Although the shoreline has gradually migrated from a water depth of ~100m–~30m, the onshore Red River has been active to produce an alternative succession of alluvial silty sand facies. This alluvial rhythm was generated in the transgressive system tract and hence it was called transgressive alluvial deposit (at TST) (Figure 7b).

In a number of boreholes conducted in the Hanoi, Hung Yen, Thai Binh, and Nam Dinh provinces, it is be clearly seen that the transgressive alluvial deposits were known to overlie directly above the eroded surface of the regressive alluvial rhythm (atTST/arLST) (Figure 5). Two rhythms of the alluvial deposits were clearly identified on high-resolution seismic sections off the Red River Delta. The transgressive rhythm is distinguished by high frequency and low amplitude of seismic reflection, showing dominant finer grained sediment over the underlying alluvial rhythm.

- *The Early-Middle Holocene transgressive coastal facies complex*, (11- 6 Ka. amt TST Q_2_^1−2^).

Within the shoreline zone limited from ~5-0m of water depth, the depositional environment has been affected by more marine process. A series of marine lithofacies have been formed and are characterized by onlapping seismic termination to produce the Q_2_^1−2^ sedimentary unit, which overlies a gray-greenish clay package of the Middle-Holocene maximum transgressive stage. This complex is composed of three facies: (1) the tidal flat sand facies: good sortness and roundness (Figure 6); (2) the estuary mud facies; and (3) the coastal swamp peat-bearing clay facies along the shoreline. These facies have been widely developed from the present coastal zone to the marginal zone of the delta and were ended when the sea level reached a maximum elevation at ~5m to produce a 3m-high marine notch on the limestone cliff in Ninh Binh (Gia Vien, Trang An). Meanwhile, a cross stratigraphy boundary between two sedimentary units was delineated: (1) a transitional facies unit aging from 12 Ka. (Based on ^14^C dating at HNK -34 borehole in Hai Hau to 7 Ka. (Based on ^14^C dating at MT-1 borehole in Hanoi and 5 Ka. (Based on ^14^C dating at VDC-25 borehole in Tam Coc) (Table 1), and (2) Early Holocene transgressive alluvial unit (atTSTQ_2_^1^).

- *The Middle Holocene (*6-5 Ka.) *maximum transgressive facies* (mt TST Q_2_^2^) is a lagoonal greenish gray clay facies covering almost the entire area of the Red River delta. It is just the marine flooding surface - the product of a thousand years of interrupted sea level before it turned to the opposite direction (Figure 7).

#### 3.2.3 Late Holocene regressive stage and evolution of the red River Delta corresponding to the highstand systems tract

The Late Holocene regressive stage (Q_2_^2−3^) of the Red River Delta was strongly controlled by the accumulation of sedimentary materials. The shoreline has migrated seaward, following three fluctuation cycles. Each cycle was marked by a paleo-shoreline zone. In the Red River Delta, three paleo-shoreline zones were dated at ~3–2.5 Ka., 1.5–1 Ka., and 0,5–0.2Ka. (Table 1, Figure 7d). Each paleo-shoreline generation is associated with two symmetrical sand ridges situated on both sides of the paleo-Red River and flower-shaped deltaic lobes.

The delta evolution has occurred under the “joint sand bars” mechanism, where sediment supply was faster than accommodation space creation. Each sand bar has been built in four stages under the impact of the three hydrodynamic elements such as the river, wave, and shore currents: (1) accumulation of sediments on the sea bottom; (2) the formation of submarine sand bars by waves and nearshore currents; (3) the submarine sand bar emerging above sea surface due to storm surge; and (4) the accretion of the sand bar and formation of tidal distributaries and mangrove swamp. By this mechanism, the delta front facies have likely been underlain by a younger deltaic plain facies, which has been formed during the last ~100–200 yrs.

### 3.3 Discussion

The Coastal zone of the Red River Delta is extended in both onshore and offshore Red River Delta, which elongates over 3 provinces: Thai Binh, Nam Dinh, and Ninh Binh. Applying sequence stratigraphy method together with lithofacies in sedimentary evolution study allowed us to elucidate the structure and distribution rules of the three systems tracts (LST, TST, HST) of the Late Pleistocene-Holocene sequence for the study area.

The lowstand systems tract (LST) is characterized by a Late Pleistocene (40 - 18 Ka.) alluvial terrigenous lithofacies group (arLSTQ_1_^3b^), which directly overlies an eroded/incised surface of the river channel. On the mainland, this facies group was identified at a depth of ~56–75m (at BH56-ND borehole in Hai Hau, Nam Dinh), ~30–50m (at BH35-TB borehole in the Ba Lat Mouth, Thai Binh), and ~15–25m (at BH-1CD borehole, in the Day river Mouth, Ninh Binh). Mineral composition of the group shows polymineral composition, poor roundness (Ro = 0.3), and is poorly sorted (So > 2.3). In the submerged area, this facies group was defined based on the interpretation of the high-resolution seismic data. The lowstand systems tract was identified by rough, hummock, low-frequency reflections representing relatively coarse grained, cross bedding channel gravelly sand facies. The lower boundary of this seismic package is a clear fluvial erosional/incised channel. It was defined as the boundary between the Late Pleistocene-Holocene sequence and the older underlying sequence (arLSTQ_1_^3b^/amhHSTQ_1_^3a^) (~40 Ka.).

The transgressive systems tract (TST) can be defined by a presence of the Early-Middle Holocene (12-5 Ka.) transgressive coastal swamp mud facies and lagoonal greenish gray clay facies group (amt, mtTSTQ_2_^1−2^). On the mainland, this group of lithofacies was observed at ~30–56m below the ground (At BH56-ND borehole); 15–30m (At BH35-TB borehole) and 5–15m (At BH1-CD) and consists of 3 lithofacies: (1) the alluvial silty sand facies of the transgressive systems tract (atTSTQ_2_^1^); (2) the coastal swamp mud facies and (3) the lagoonal greenish, bluish gray clay facies. Off the red River Delta, this group of facies was clearly identified on high resolution seismic profiles, which are distinguished by 3 different seismic reflection configurations: (1) Rough and chaotic reflection representing the gravelly sand facies; (2) Discontinuous, low-amplitude reflection associated with tidal flat cross bedding mud facies deposited in low energy regime; (3) Horizontal smooth reflection showing the marine flooding plain clay facies. The age of the upper boundary was defined as 5 Ka.

Highstand systems tract (HST): The onshore HST was subdivided into two groups of lithofacies: (1) Middle-Late Holocene (5–2.5 Ka.) burial submarine deltaic clayish silt facies aged; (2) Late Holocene (2.5 Ka. -Present) deltaic plain sandy silt facies group. This lithofacies group consists of 2 main facies: river mouth sand bar facies, characterized by high quartz content (>85%), good sortness, and roundness (So_mean_ = 1.3; Ro_mean_ = 0.6) and deltaic floodplain clay silt facies. Meanwhile, the offshore HST clearly shows divergent seismic reflection, typical for the modern delta front and prodelta, which extends from 0m to 20m of water depth (amhHSTQ_2_^3^). Within the 20–30 m of water depth zone, it is characterized by horizontal, parallel seismic reflection, typical for shallow marine mud and sand facies (mhQ_2_^3^).

Stratigraphy of the Red River Delta has been introduced by various Vietnamese Quaternary geologists (Ky HN, 1973; Thang, 1996) and the Quaternary sediments in the coastal zone of the Red River Delta was subdivided into 5 Formations: (1) the Early Pleistocene Le Chi Formation (Q_1_^1^lc); (2) the Middle-Late Pleistocene Hanoi Formation (Q_1_^2−3^hn); (3) The End of the Late Pleistocene Vinh Phuc Formation, (Q_1_^3b^vp); (4) The Early-Middle Holocene Hai Hung Formation (Q_2_^1−2^hh) and (5) The Late Holocene Thai Binh Formation (Q_2_^3^tb). Although this stratigraphical division has long time been used for various purposes such as geological mapping and mineral resources survey, it is uncorrelatable to the central and southern coastal plains of Vietnam as well as to the continental shelf because of the following reason: (1) the difference in the number of classified formation and ages between the Red River Delta and other regions; (2) the authors used different local names denoted to each formation; (3) criteria for classification introduced by authors are different. The above-mentioned challenges are attributed to: (1) The lack of reliable data; (2) New research methods have not been applied, especially the sequence stratigraphy method. In such context, the authors must have relied on the visible description of sediments for dividing sedimentary formation. The previous authors divided the End of the Late Pleistocene-Holocene coastal zone of the Red River Delta into 3 formations: (1) the Vinh Phuc Formation (Q_1_^3b^) based on the presence of the "mottled clayish silt" layer; (2) Hai Hung Formation (Q_2_^1−2^) based on the presence of greenish gray clay layer. The boundary between the End of the Late Pleistocene and the Early Holocene (Q_1_^3b^/Q_2_^1^) was picked at 10 Ka. by the authors of this work and (3) the Thai Binh formation (Q_2_^3^) was established for the upper youngest deposit and the age of the Late/Middle Holocene boundary was determined as 3 Ka. These boundaries were not actually identified in sedimentary geological sections in the coastal zone of the Red River Delta. In this paper, the authors only contribute to revising the stratigraphic classification scheme and attempt to reconstruct the Late Pleistocene - Holocene sedimentary evolution of the Red River Delta in relation to the sea level change and tectonic activities. The results of this paper encourage geoscientists to apply the sequence stratigraphy in studying the Quaternary geology of the Red River Delta and its adjacent coastal zone as it is a sensitive transition between the land and the sea, which is important not just for natural resources but also for human residence and ecosystem sustainability.

## 4 Conclusions

Our study results mentioned above allow us to draw the following conclusions:

1. The Late Pleistocene - Holocene sediments in the coastal zone of the Red River delta have been resulted from a complete sequence of three systems tracts:
  - The lowstand systems tract (LST) is characterized by a complex of the Late Pleistocene (arLSTQ_1_^3b^) alluvial silty sand present in two facies: (1) The lower facies consists of fluvial channel polymineral sand facies with poor sortness and roundness and (2) the upper facies is a flood plain silty clay facies.
  - The transgressive systems tract is characterized by the Late Pleistocene - middle Holocene (at, amt, mtTST Q_1_^3b^-Q_2_^1−2^) transgressive alluvial facies, the coastal marsh facies complex, and the lagoon greenish-gray clay facies. The transgressive alluvial facies group (atTST) is also similar to the regressive alluvial facies group. However, this facies group demonstrates the thinner section and finer grain size. The transitional facies group includes three facies (amtQ_1_^3b^-Q_2_^1−2^TST): (1) The tidal flat sand facies contains lateritic gravelly sand and shell fragments; the origin of these lateritic material has been resulted from oxidization weathering process to produce iron hydroxide (Fe_2_O_3_.nH_2_O) present in mottled silty clay layer, whose age varies from the Early to Late Pleistocene (Q_1_^3a^); (2) the coastal swamp peat-bearing clay facies; and (3) the tidal channel mud and estuary sandy mud facies. By the end of this period, the maximum transgressive stage has led to the formation of the lagoonal greenish grey clay facies.
  - The highstand systems tract is distinguished by a deltaic clayish silt facies complex (amhHSTQ_2_^2−3^), which is composed of three facies groups: (1) the modern alluvial clayish silty sand facies group; (2) the burial deltaic clayish silt facies group; and (3) the submarine deltaic sandy mud facies group (0–25 m deep).
2. The evolution of the Late Pleistocene - Holocene sediments of the Red River delta has brought about the formation of different lithofacies complexes and the patterns of the crossed boundaries in the stratigraphy. On the surface of the coastal zone, there have been two different facies complexes: the upper modern delta plain clayish silt and sand ridge facies complex and the lower modern delta plain mud facies complex. Above this section, two facies complexes of the coastal zone have been defined: (1) the burial submarine deltaic facies complex and (2) the deltaic plain facies complex.
3. Determination of the Late Pleistocene - Holocene stratigraphy of the coastal zone of the Red River Delta was approached by using sequence stratigraphy framework with three systems tracts (LST, TST, and HST) and three time-domain cross boundaries accordingly: (1) the regressive boundary (LST) lasted from 40 to 18 Ka; (2) the transgressive boundary (TST) from 18 to 5 Ka.; and (3) the highstand boundary from 5 Ka. to Present.

## Declarations

### Author contribution statement

Hoang Phan Hải Yen, Tran Thị Thanh Nhan, Tran Nghi: Conceived and designed the experiments; Analyzed and interpreted the data; Wrote the paper.

Ngo Quang Toan, Hoang Anh Khien, Doan Dinh Lam: Contributed reagents, materials, analysis tools or data; Wrote the paper.

Hoang Van Long, Dinh Xuân Thanh, Nguyen The Hung, Nguyen Thị Huyen Trang: Analyzed and interpreted the data; Wrote the paper.

Tran Ngọc Dien, Nguyen Thị Tuyen, Tran Xuan Truong, Tran Thị Dung, Nguyen Thi Phuong Thao, Vu Quang Lan: Performed the experiments.

### Funding statement

This work was supported by the Ministry of Science and Technology, the National Key Research Program KC.09/16–20 (grant number KC.09.02/16-20) from Vietnam; the VNU Asia Research Center, the Korean Foundation for Advanced Studies (grant number CA.17.10A); and 10.13039/100015547VNU University of Science, Vietnam.

### Data availability statement

The authors do not have permission to share data.

### Declaration of interests statement

The authors declare no conflict of interest.

### Additional information

No additional information is available for this paper.

## References

[bib31] Funabiki A., Saito Y., Phai V. Van, Nguyen H., Haruyama S. (2012). Natural levees and human settlement in the Song Hong (Red River) delta, northern Vietnam. Holocene.

[bib1] Hall R. (1998). The plate tectonics of Cenozoic SE Asia and the distribution of land and sea.

[bib2] Hanebuth T.J.J., Saito Y., Tanabe S., Vu Q.L., Ngo Q.T. (2006). Sea levels during late marine isotope stage 3 (or older?) reported from the Red River delta (northern Vietnam) and adjacent regions. Quat. Int..

[bib3] HutChison C.s. (2014). Tectonic evolution of Southeast Asia. Bull. Geol. Soc. Malays..

[bib4] Khien H.A. (2003). Quaternary Geological Map at Scale of 1/50000 Hung Yen - Phu Ly Area.

[bib5] Ky H.N. (1973). Geological Map of Thai Binh – Nam Dinh, scale:1/200.000.

[bib6] Ky H.N. (1978). Results of C14 Radiometric Dating of Quaternary Geology of Bac Bo plain.

[bib7] Lam D.D. (2003). Holocene Sedimentary Evolution in the Red river delta.

[bib8] Li Z., Saito Y., Matsumoto E., Wang Y., Tanabe S., Lan Vu Q. (2006). Climate change and human impact on the Song Hong (red River) delta, Vietnam, during the Holocene. Quat. Int..

[bib9] Lieu N.T.H. (2006). Holocene Evolution of the Central Red River Delta, Northern Vietnam, Lithological and Mineralogical Investigations.

[bib10] Mathers S., Zalasiewicz J. (1999). Holocene sedimentary architecture of the red River delta, Vietnam. J. Coast Res..

[bib11] Mien Q.N., Phon K.L. (2000). Some results of C14 dating in investigation on Quaternary geology and geomorphology in Nam Đinh - Ninh Binh area. Viet Nam. J. Geol. B/.

[bib12] Nghi T. (2003). Sedimentology.

[bib13] Nghi T. (2012). Sedimentology.

[bib14] Nghi T. (2018). Sedimentary Geology of Vietnam.

[bib15] Nghi T., Toan N.Q. (1991). Characteristics of sedimentary cycles and Quarternary geological evolution of Red River delta. J. Gelogy No.

[bib16] Nghi T., Ngo Quang Toan, Do Thi Van Thanh, Nguyen Dinh Minh, Nguyen Van Vuong (1991). Quaternary sedimentation of the principal deltas of Vietnam. J. Southeast Asian Earth Sci..

[bib17] Nghi T., Nhuan M.T., Ngoi C. Van, Utrecht P., van Weering T.C.E., van den Bergh G.D., Thanh D.X., Nguyen N.D., Phai V. Van (2002). Holocene Sedimentary Evolution, Geodynamic and Anthropogenic Control of the Balat River Mouth Formation (Red River Delta, Northern Vietnam).

[bib18] Nghi T., Nhuan M., Van Ngoi C., Van Dai N., Xuan Thanh D., Dinh Nguyen N., Nguyen-Thanh L., Dam Q.-M., Quang Toan N. (2003). GIS and Image Analysis to Study the Process of Late Holocene Sedimentary Evolution in Balat River Mouth, Vietnam.

[bib19] Nghi T., Tuyen N.T., Thanh D.X., Nguyen N.D., Nhan T.T.T., Thai N.D., Trang N.T.H. (2016). Paleoshore and cross boundary of late Pleistocene – Holocene systems tract in the north and middle north. J. Geol..

[bib20] Nghi T., Tuyen N.T., Thanh D.X., Nguyen N.D., Nhan T.T.T., Thai N.D., Trang N.T.H., Chuan L.V., Long N.H. (2017). Paleo-lithofacies and geographic characteristics of late Pleistocene – Holocene in Ba Lat mouth area. J. Mar. Sci. Technol..

[bib21] Rukhin L. (1969). Basic Sedimentology.

[bib22] Tan M.T., Dung L. Van, Bach L.D., Bieu N., Nghi T., Long H. Van, Huong P.T. (2014). Pliocene–Quaternary evolution of the continental shelf of central Vietnam based on high resolution seismic data. J. Asian Earth Sci..

[bib23] Tanabe S., Hori K., Saito Y., Haruyama S., Quoc Doanh L., Sato Y., Hiraide S. (2003). Sedimentary facies and radiocarbon dates of the Nam Dinh-1 core from the Song Hong (red River) delta, Vietnam. J. Asian Earth Sci..

[bib24] Tanabe S., Hori K., Saito Y., Haruyama S., Vu V.P., Kitamura A. (2003). Song Hong (Red River) delta evolution related to millennium-scale Holocene sea-level changes. Quat. Sci. Rev..

[bib30] Tanabe S., Saito Y., Lan V.Q., Henebuth Till J.J., Lan N.Q., Kitamura A. (2006). Holocene evolution of the Song Hong (Red River) delta system, northern Vietnam. Sediment. Geol..

[bib25] Thang N. (1996). Geological and Mineral Maps in Thai Binh – Nam Đinh, Scale: 1/50.000.

[bib26] Toan N.Q. (1989). Geological Map of Hanoi, scale1/50.000.

[bib27] Trask P. (1932). Origin and Environment of Source Sediments of Petroleum.

[bib28] Vietnam oil and gas Group (2007). Geology and Petroleum Resources of Vietnam.

[bib29] Zahirovic S., Seton M., M R.D. (2014). The cretaceous and cenozoic tectonic evolution of Southeast Asia. Solid Earth.

